# Social capital and its role to improve maternal and child health services in Northwest Ethiopia: A qualitative study

**DOI:** 10.1371/journal.pone.0284592

**Published:** 2023-04-21

**Authors:** Endalkachew Worku Mengesha, Gizachew A. Tessema, Yibeltal Assefa, Getu Degu Alene

**Affiliations:** 1 Department of Reproductive Health and Population Studies, School of Public Health, College of Medicine and Health Sciences, Bahir Dar University, Bahir Dar, Ethiopia; 2 Curtin School of Population Health, Curtin University, Perth, WA, Australia; 3 School of Public Health, The University of Queensland, Brisbane, Australia; 4 Department of Epidemiology and Biostatistics, School of Public Health, College of Medicine & Health Sciences, Bahir Dar University, Bahir Dar, Ethiopia; Moi University School of Arts and Social Sciences, KENYA

## Abstract

**Background:**

Social capital is a set of shared values that allows individuals or groups receive emotional, instrumental or financial resources flow. In Ethiopia, despite people commonly involved in social networks, there is a dearth of evidence exploring whether membership in these networks enhances uptake of maternal and child health (MCH) services. This study aimed to explore perspectives of women, religious leaders and community health workers (CHWs) on social capital to improve uptake of MCH services in Northwest Ethiopia.

**Methods:**

We employed a qualitative study through in-depth interviews with key informants, and focus group discussions. A maximum variation purposive sampling technique was used to select 41 study participants (11 in-depth interviews and 4 FGDs comprising 7–8 participants). Data were transcribed verbatim and thematic analysis was employed using ATLAS.ti software.

**Results:**

Four overarching themes and 13 sub-themes of social capital were identified as factors that improve uptake of MCH services. The identified themes were social networking, social norms, community support, and community cohesion. Most women, CHWs and religious leaders participated in social networks. These social networks enabled CHWs to create awareness on MCH services. Women, religious leaders and CHWs perceived that existing social capital improves the uptake of MCH services.

**Conclusion:**

The community has an indigenous culture of providing emotional, instrumental and social support to women through social networks. So, it would be useful to consider the social capital of family, neighborhood and community as a tool to improve utilization of MCH services. Therefore, policymakers should design people-centered health programs to engage existing social networks, and religious leaders for improving MCH services.

## Background

Social capital is considered to be a cross-cutting determinant of health since the World Health Organization’s Commission for Social Determinants of Health was launched in 2010 [1]. Despite there is no single definition to date, in most public health research, the definition provided by Robert Putnam is mostly adopted, according to this definition, social capital refers features of social organization such as networks, norms, and social trust that facilitate coordination and cooperation for mutual benefit [2]. It provides individuals and groups with access to resources and supports by virtue of membership in social networks [3, 4]. The concept of social capital has structural and cognitive dimensions that are not mutually exclusive and characterized in terms of social relations as what people ‘do’ and what people ‘feel’, respectively. The structural dimension refers to the intensity of an individual’s participation in community networks measured in objective terms. The cognitive dimension involves subjective aspects such as trust, values, attitudes and perceptions of an individual’s social network [5, 6]. On the other hand, it was also viewed as bonding and bridging. Bonding social capital is inward looking and involves those who share particular social outlooks or characteristics such as family and close friends. Bridging social capital consists of those who are different in their social outlook. Interacting with diverse people likely increases the chances of sharing new information. Moreover, the concept of social capital has been conceptualized and measured in four main ways: i) personal relationships; ii) social network support; iii) civic engagement; and iv) trust and cooperative norms [7]. Social network refers a structure composed of a set of actors in which members are connected by one or more relations [8]. The networks could exist between persons, communities, organizations, or nations. It provides access to various forms of social capital such as information, social support, and values along with economic resources [9–11].

In Ethiopia, women and family members are involved in social networks including *‘Iddir’*, *‘Iqqub’*, *‘Maheber’ and ‘Senbetie’* that could be great opportunity for them to receive important health information and necessary social and financial support. *‘Iddir’* is a self-help voluntary association that serves as economic and social insurance at times of death and other crises. Households become members of the associations and pay fixed monthly contributions [12]. *‘Iqqub’* is a circular saving system in which relatives, neighbours or friends collect money to build each member’s financial capacity. *‘Maheber’ and ‘Senbetie’* are socio-religious associations that hold gatherings, with spiritual and social functions named after saints [13–15]. (S1 Appendix) Previous studies reported social capital as one of the social determinants of health [16, 17].

Ethiopia is committed to achieving universal health coverage through the strengthening of primary health care [18]. The Ethiopian government has been implementing different community health programs as part of the Health Extension Package adopted in 2004 to improve the provision of essential health services including MCH service. The government launched its response through two programs: the Accelerated Expansion of Primary Health Care Coverage, and the Health Extension Program (HEP). As part of the programs, CHWs including Health Extension Workers (HEWs), and Women Developmental Army (WDA) are highly involved in providing promotive and preventative health services [19–21]. The combined effect of the WDA and HEWs increased uptake of MCH services [22, 23]. The HEP has improved the utilization of health services by linking communities and health facilities, particularly health centers. However, the program is currently facing challenges related to productivity and efficiency of HEWs; working and living conditions of HEWs; capacity of health posts; and social determinants of health [24]. Despite consistent implementation of these approaches, the MCH service coverage for Ethiopia was low and varies with geographical location.

Maternal and child health services such as antenatal care, childbirth at health facility and postnatal care and immunization services are critical for preventing and reducing maternal and child mortality and morbidity [25, 26]. However, women and children in low- and middle-income countries (LMICs) often have limited access to these essential health services [27]. The health services uptake is challenged by several factors including low quality health systems, socio-demographic and economic factors such as rural residence, lower maternal age, education and household wealth; lack of women’s voice in the decision-making process, and poor preference of childbirth at a health facility. The health services uptake is challenged by several factors including low quality health systems [28], socio-demographic and economic factors such as rural residence, lower maternal age, education and household wealth [29–35]; lack of women’s voice in the decision-making process [36], and poor preference of childbirth at a health facility [37]. In this study, MCH services refer health services provided to women and children during pregnancy, intrapartum, and postpartum periods. Particularly, antenatal care (ANC), health facility delivery, postnatal care and childhood immunization were considered. Evidence showed that individuals with more social capital have better health and live longer [38, 39]. Social capital positively influences health behavior in addition to economic and political systems. For example, the decline of social capital in USA led to many problems. While in Italy, membership to civic organizations enhances particularized and generalized trust among and between community members. A study conducted in Africa, Asia and Latin America also showed that health care services are conceptually grounded based on nuclear family model in which the role of women and men are overlapping and grandparents often not nearby [40, 41]. However, in practice, family members, social networks and older women in the community have their own cultures and strategies to promote uptake of MCH services [36, 41, 42].

Systematic reviews conducted in LMICs showed that the socio-cultural context influenced uptake of MCH services. The social and cultural norms in a given society and perceived standards of acceptable attitudes and behaviors within formal and informal networks are connected to uptake of MCH services in several of ways. For example, women may form opinions and engage in specific behaviors that could negatively affect their health and the health of their newborn because of perceived consequences of not conforming to social norms and how others in their social network are behaving. In addition, lacks of social interaction, mistrust towards the existing health service, healthcare providers or institutions, peer influence and conformity with norms have been shown to be associated with low MCH service utilization [14, 43–45]. In Ethiopia, maternal health care service seeking of women for attending institutional delivery (28.7%) and postnatal care (22.6%) were low. Low MCH services uptake is attributed to socio-demographic and cultural factors [46, 47] and social capital could have some value to improve access but no previous studies has explored them [48]. Other countries’ social networks such as Harambe in Kenya found to improve healthcare services uptake [49]. However, despite there are a range of active social networks in Ethiopia, [46, 50, 51], there is a paucity of evidence in exploring women’s and community’s perspectives whether or not these networks enhance women’s access to health services. Hence, this study aimed to explore perspectives of women, religious leaders and CHWs on the influence of social capital in improving uptake of MCH services in Northwest Ethiopia. The finding from this study could inform policymaker on how to consider individual, family, neighbourhood and community social capital in MCH programs.

## Methods

### Study setting

This study was conducted in South Gondar Zone, Amhara region, Ethiopia which comprises of 13 rural districts and 8 town administrations with a total of 404 *Kebeles* (the smallest administrative unit in Ethiopia). The center of the Zone–Debre Tabor town–is located 756 km away from Addis Ababa (the capital city of Ethiopia). The main ethnic group reported in South Gondar was the Amhara (99.7%); all other ethnic groups made up 0.3% of the population. Amharic was spoken as a first language by 99.7%; the remaining 0.3% spoke all other primary languages. Majority (96.14%) of the population practiced Ethiopian Orthodox Christianity, and 3.68% were Muslim [52]. Based on the 2022 Amhara regional health report, the Zone has a total population of 2,651,350 people, of whom 23% were women in reproductive age group (15–49), and 3.37% of them were pregnant. There are 608,713 households in the zone with an average of 4.36 persons per household. There are one comprehensive specialized hospital, eight primary hospitals, 96 health centers and 408 health posts serving the community [53]. Maternal and child health services are provided at all levels of healthcare. Regarding the coverage of MCH services in Amhara region, 82.6%, 55.7% and 39.8% women were received ANC, delivery and PNC from a skilled provider in 2019, respectively [54].

### Study design, participants and recruitment

A phenomenological qualitative study was employed to explore the experiences and perspectives of women, community leaders and community healthcare providers.

In this study, participants were selected using the maximum variation purposive sampling technique to get greater insights into a phenomenon by looking at it from different angles. In-depth interviews were conducted with HEWs and religious leaders. In addition, postpartum women and WDA members participated in FGDs, in separate sessions. A total of 41 study participants (eight postpartum women, five HEWs, 22 WDA leaders, and six religious leaders) were included in this study. Participants were recruited from different *Kebeles* in Debre Tabor, Wereta, Estie and Dera districts. The inclusion of participants from different geographical areas allowed for capturing the local sociocultural, religious and gender norms that might influence uptake of MCH services.

Prior to the commencement of the FGD, participant information such as their demographic characteristics, and comprehensive list of social networks they participated at the time of pregnancy or in the postpartum period was requested separately from each participant. In the group discussion, participants were invited to share experiences how they are connected, their frequency of contact, interactions with the network members, relationship with network members, and whether members in a network have known their pregnancy or childbirth status. Participants’ perceptions and perspectives on whether involvement in any of the social network helped them to use MCH services were also part of the discussion. To assess the function of social network, women were asked about how each network member was engaged in their pregnancy or childbirth experiences; the kind of support or influence each member could have on women to use MCH services. Likewise, key informants were asked to identify roles of individuals involved in health decisions about women’s pregnancy; describe various supports of family members, WDA, HEWs, community and religious leaders provide to postpartum women that could facilitate use of MCH services.

### Data collection process

Data collection was carried out using semi-structured in-depth interview and focus group discussion (FGD) guides (S2-S5 Appendices) that were pretested before data collection. The lead author (EWM) conducted in-depth interviews and moderated FGDs. Data were collected in local language (Amharic) until new information has no longer appeared in subsequent interviewees and discussants. All the interviews and FGDs were audio-recorded. In addition, note taker (MF) took field notes to document their impressions. The data collection was conducted between November 25, 2021 and May 07, 2022.

### Data management and analysis

Upon completion of each interview, the audio recording was transcribed into the Amharic language, and these transcripts were then translated into English by the primary investigator and made available in Microsoft word files for analysis. Before analysis, audio-recorded in-depth interviews and FGDs have been listened to carefully. Although the process of data transcription was time-consuming, it allowed us to familiarize ourselves with the data.

The transcribed and translated data were then exported to ATLAS.ti software for data management and analysis. Data were analyzed using Braun and Clark’s inductive thematic analysis approach. An inductive approach refers the identified themes are strongly linked to the data themselves and then move to broader generalisations [55]. Before the commencement of coding, EWM read and re-read transcripts repeatedly to shape ideas and identification of possible patterns. We adopted a basic interpretive qualitative approach, focusing on uncovering meaning from research participants’ views. After familiarizing ourselves with the data, all data have been initially coded and collated, and a long list of the different codes was generated. Then, we re-focused the analysis at the broader level of themes, rather than codes, which involves sorting the different codes into potential themes, and collating all the relevant coded data extracts within the identified themes. In addition, a final version of the data-driven codes, sub-themes and themes was discussed with GAT and YA.

### Trustworthiness

To maintain trustworthiness of the data, multiple data sources such as HEWs, religious leaders, WDA and postpartum women were included. In addition, triangulation of two types of data collection methods (in-depth interview and FGDs) could ensure credibility of the findings. To ensure accuracy, sample of transcripts were randomly checked against the original recordings. Four debriefing sessions for the research team members were prepared to refine the in-depth interview and FGD guides, data collection process, identify saturation of data, and discuss key findings.

### Ethical considerations

Ethical clearance was obtained from Institutional Review Board (IRB) of CMHS, Bahir Dar University, Ref No. CMHS/IRB/331/2021. Support letters were also obtained from the Amhara Public Health Institute and Woreda health offices. To keep the confidentiality of the study subjects, any personal identifiers were not asked in the interviews and FGDs. Verbal consent was obtained from all participants based on the knowledge that many women in rural Ethiopia are not able to give written consent, the study was not likely to be harmful and all study participants were over the age of 18. Privacy and confidentiality was maintained throughout the data collection, analysis and presentation.

## Results

### Characteristics of study participants

Forty-one study participants, 11 in-depth interviews with key informants and four FGDs with 30 women (each comprising 7–8 individuals), were employed. Majority of key informants were educated, college and above. Out of the 29 postpartum women who have children, 14 of them gave birth in hospital. (Tables 1 and 2)

**Table 1:** Socio-demographic characteristics of in-depth interview participants.

Variables	Categories	In-depth interview (n = 11)
**Sex**	Male	6
	Female	5
**Religion**	Orthodox	9
	Muslim	2
**Occupation**	Priesthood	4
	Head of Islamic affairs	2
	Health extension worker	5
**Educational level**	Able to read and write by religious education	2
	Primary (grade 1–8)	1
	College and above (12+)	8

**Table 2:** Socio-demographic and reproductive health characteristics of FGD participants.

Variables	Categories	FGDs (n = 30)
**Residence**	Urban	23
	Rural	7
**Marital status**	Single	3
	Married	25
	Divorced	2
**Occupation**	Housewife	18
	Farmer	6
	Merchant	4
	Government employee	2
**Educational level**	Never attended	5
	Primary (grade 1–8)	15
	Secondary (grade 9–12)	6
	College and above (12+)	4
**Place of delivery for last pregnancy**	Home	2
	Health post	1
	Health center	12
	Hospital	14

### Identified themes and sub-themes

In this study, four overarching themes and 13 sub-themes of social capital were identified that could influence the uptake of MCH services. The identified themes include social networking, social norms, community support, and community cohesion. (Table 3)

**Table 3:** The identified themes, and sub-themes.

Themes	Sub-themes
**Social networking**	Group membership
	Social participation
	Social organizations and linkages
	Community mobilization
**Social norms**	Traditional practice
	Social norms
	Religious value
**Community Support**	Social support
	Emotional support
	Financial support
**Community cohesion**	Domestic cohesion
	Neighbourhood cohesion
	Trust

#### Social networking

Most women, HEWs, WDA and religious leaders reported that they were participating in social networks such as ‘*Iddir*’, *‘Iqqub’* or ‘*Maheber’*. Women participated in mother’s forum, women’s association, pregnant women conference, group ANC, and other volunteer organizations. In the absence of men, more women could talk openly.

*“I am a member of associations called ‘Iddir’*, *‘Iqqub’ and ‘Maheber’*. *On that forum*, *we freely discuss it [available health services for women and children]*.*”* (a 35-year old HEW)

The other participant in the FGD highlighted that,

*“I am a member in a union*. *It is a good opportunity to talk about women and children on association*.*”* (FGD, a 26-year old WDA leader)

In addition, most religious leaders were participating in different groups including developmental groups, community dialogues, and peace and security counseling groups.

*“We are members of the Kebele development team; we work together*. *In the Kebele*, *there is women’s structure where they can participate*.” (a 36-year old religious leader)

*“I have been the head of Islamic affairs for 30 years and the chairman of ‘Iqqub’ for 42 years*.*”* (a 78-year old religious leader)

*“There is a volunteer organization in our Kebele*. *Along with the clergy*, *I am the deputy district commissioner and ‘Mergeta’ [a religious leader in Orthodox Church] is the chairperson of peace council*. *In collaboration with it*, *I became involved in promoting maternal health activities*.*”* (a-54 year old religious leader)

Some social networks had regular meeting schedules and that facilitated the group members to access health information. Then, they could transfer messages to their neighbours.

*“‘Iqqub’ and ‘Iddir’ have their own establishment purpose*. *In our area*, *‘Iqqub’ is associated with saving money; in the same way*, *‘Iddir’ is valuable when a person dies*. *Health extension workers could use these social networks to create awareness*.*”* (a-36 year old religious leader)

In addition, some HEWs indicated that they leveraged the available social networks to create awareness.

*“They [women] have a conference once a month*. *It is a place where only women can discuss*. *The women who come here could be from any Kebele; they talk and exchange experiences*.*”* (a 32-year old HEW)

Although HEWs recognized that the social networks and its members favor the community-based healthcare activities provided by them, few religious leaders, *‘Iddir’* and *‘Iqqub’* members had no close relationship with them.

*“If we organize our work*, *we can easily get the job done*. *We often meet those who are close to us; we have little contact with ‘Iqqub’ [leaders]*. *The religious fathers and ‘Iddir’ [leaders] are not closer to us*.*”* (a 32-year old HEW)

*Social organizations and linkages*. Social organizations and networks were helpful platforms to mobilize the community and transmit essential health messages to individuals who interrupted their children’s vaccination and traced through the social networks. There are 1 to 5 groups (i.e.one women lead a group of five women), *‘Iddir’* and *‘Iqqub’*; and using these networks, variety of health information could have been provided to the community. Community health workers provided health information and regular messages in the *‘Iddir’*, *‘Iqqub’* or in the church on Sunday morning time.

*“Social organizations are very important; we believe that they are the solutions for ill-health and other issues*. *Both ‘Iqqub’ and ‘Iddir’ are good social networks*.*”* (a 54-year old religious leader)

*“In ‘Iddir’*, *we find large members*, *and an opportunity to explain about [the importance] of immunization services and sanitation*. *For example*, *I can personally go and send messages to two or three social organizations for women and children to use health services*. *People who did not attend that meeting will hear the information using their social networks*.*”* (a 32-year old HEW)

In addition, HEWs explained that MCH service provisions could be improved by using social networks.

*“We can further improve women’s and children’s health services using social networking*. *Instead of going from house to house*, *it is better to go to a place [‘Iddir’] where you can access many people*. *When we go from house to house*, *we find a mother or a child; we may not get the whole family*. *If we are creating awareness where the community is gathered; I think it [‘Iddir’s meeting] will be better*.*”* (a 35-year old HEW)

*“People know the care needed for women and babies; we need them [members in social networks] if someone has discontinued vaccination; we can resume vaccination provision by identifying them in ‘Iddir’*.*”* (a 32-year old HEW)

*Community mobilization*. Health professionals, nongovernmental organizations and religious leaders mobilize the community and create awareness about the health of women and children using various platforms. However, continuous community mobilization was needed. The district and *Kebele* administration used a megaphone for promoting the messages.

*“I have done nothing alone*, *however*, *I involve in community mobilization together with them*. *But*, *the society always needs an advertising campaign*.*”* (a 36-year old religious leader)

*“Sometimes nongovernmental organizations mobilize us; at that moment*, *we help each other*. *We also encourage our spiritual children to learn and to be vaccinated on time; sometimes people associate vaccination with something else and some people think it hurts*. *We try to make those people aware*.*”* (a 42-year old religious leader)

*Social participation*. As most religious leaders and HEWs highlighted, women are participating in various social networks. The connection among members was a good opportunity that would help to gain financial support, health information, and to exchange ideas one another. This was attested by a religious leader:

*“I participate in ‘Iqqub’*, *‘Iddir’ and associations*. *I have also been a Moses [chairperson] of the association*. *I think my social interaction with my friends is good*. *We meet at church in Tsig`e [Sabbath] association every week*, *and sometimes*, *we meet 2–3 times a week because we live close by*.*”* (a 36-year old religious leader)

*“As a health extension worker*, *the connection between the family and the community will minimize my workload when I meet them together*, *instead of meeting them one by one*. *It is good to get together as it allows sharing one’s insights with one another; to provide health education*, *and conduct drama conversations*.*”* (a 30-year old HEW)

#### Social norms

The religious thought, belief and value of women and religious leaders as well as the social norm, traditional practices and culture of a community could influence uptake of MCH services.

This was reflected by a religious leader:

*“In the absence of men*, *more women can talk openly*. *It is easier for women to tell women than men and these women’s forums are important…*. *It is well known that women are often marginalized in our society*. *But*, *now it is improving*, *especially in the city*. *I think there are some associations that undermine women; I think it [societal belief] will have a negative effect on maternal service utilization*.*”* (a 36-year old religious leader)

Although social groups did not engage directly in the discussion of MCH services, the neighbours are concerned about young girls in the community who feel scared to visit facilities for pregnancy care. When HEWs visit house to house, the community itself tells information about those adolescents that hide their pregnancy and are ashamed of beginning the care.

*“During my discussion*, *they [neighbours] informed me that there is someone pregnant in their village*. *It is not acceptable for someone to get pregnant out of wedlock*. *We meet that young woman [who reported to be pregnant] through them and get her started [pregnancy care]*.*”* (a 32-year old HEW)

The previous practice of women seeking maternal care services from the health center or hospital was low. Fear for lack of privacy of women and the perception of the community towards adolescent pregnancy were mentioned as reasons for not commencing ANC booking on timely basis. Some women also described that giving birth at health facility is not comfortable for them in which delivery coach might expose their private bodies.

*“They [Women] did not go to the health center since there is a sense of shame*. *They are not very happy to show their bodies*.*”* (a 32-year old HEW)

Other participants had similar viewpoints,

*“If some of them were pregnant from relatives or during adolescent time*, *there is a fear to express their feelings*. *They are obsessed with it*, *‘how can I reveal my secret*?*’”* (a 36-year old religious leader)

*“There is something shameful about pregnancy with young people because they suddenly become pregnant out of our culture*. *They have a fearful attitude*, *they just hide; especially those who are from rural areas*. *But*, *health extension workers teach well*, *and create awareness*.*”* (a 38-year old religious leader)

In addition, most religious leaders condemned abortion and adolescent pregnancy without marriage. They explained that abortion after one month or two months of pregnancy is just considered killing a human being.

*“There are young people who are giving birth and drop anywhere*. *They called abortion*. *When we teach in the church*, *we strongly condemn this*. *In theology*, *this is a creation of God*, *it is God’s building and we do not support abortion services*.*”* (a 38-year old religious leader)

There is remarkable difference among rural and urban communities in uptake of MCH services and men’s involvement in it. Unlike women living in rural area, most urban women were educated and understand the importance of MCH services in which they gave birth in health facilities. Besides, most of the husbands in the city support their wives.

*“As a city*, *there is no much problem as far as I can see*. *In fact*, *most of the men in the city support their wives and come together for vaccinations*. *Other family members and neighbours are also come along them; it’s good to see such things*. *In my opinion*, *family relationships are good*.*”* (a 32-year old HEW)

However, traditional practices and perceptions of the community in rural settings could be barriers to women giving birth in health facilities. Women are also affected by workload and food shortages.

*“There are many problems when we go to the rural area*. *Some traditional practices*, *such as giving birth out of home or in health facility may be bad for the mother and a baby*. *You will find communities that think that she will strike by Satan*. *They believe that when she gives birth*, *she sprays her blood*.*”* (a 30-year old HEW)

#### Community support

*Social support*. The community had a culture to provide support for women when there was no ambulance and get difficult to travel to the health facility. Most women get social support from neighbours and *‘Iddir’* while they were in trouble or family grief.

*“If a woman is seriously ill in the evening*, *there is no ambulance and is difficult to travel to the health facility in the dark*. *If the neighbourhood relationship is good*, *the neighbour will make a transport bed for her*. *Then*, *she will be taken to health facility by carrying via local bed*. *Likewise*, *we take her to health facility in the evening when her labor started*. *This is how social life is important*.*”* (a 42-year old religious leader)

*“Any society has a responsibility to support women*. *For example*, *if I find a mother falling out of the street*, *I immediately call an ambulance*, *a Bajaj*, *or a person to bring her to the health facility*.*”* (a 38-year old religious leader)

Majority of poor women received material, food and financial support from neighbours, religious groups and university students. Religious fathers coordinate wealthy people and the community to feed the poor, and dress clothes for the naked.

*“…After giving birth*, *the neighbour said*, *‘she has nothing to eat or drink*.*’ we support her by buying clothes*. *For example*, *in our Orthodox religion*, *a boy is baptized at 40 days old and a girl baptized at 80 days of old*. *If she is poor and has no money*, *we will buy clothes for her ceremony*.*”* (a 38-year old religious leader)

*Emotional support*. Religious leaders along with HEWs encourage women to give childbirth in a health facility; clarify misperception of people towards vaccinations; the need to respect pregnant women, and give proper attention to children. The WDA leaders had a list of pregnant women in the *Kebele* and report to HEWs every two weeks.

*“There is an idea support; no one supports money because everyone knows what money is (laughing)*. *However*, *there is emotional support by counseling and encouragement*.*”* (a 38-year old religious leader)

*“Religious leaders are especially helpful because they are not supposed the notion of conveying bad things in the community*. *They are very useful to us because they are respected by the community*.*”* (a 30-year old HEW)

*"Our main role is to provide advice to women on vaccination and I provide counseling for pregnant women*, *at least twice a month*. *We recommend women to eat nutritious vegetables and fruits*.*”* (FGD, a 33-year old WDA leader)

*Financial support*. A social network such as ‘*Iddir*’ was one of financial support mechanisms for the community. When children get sick, they [families] borrow money from *‘Iddir’* and save the life of them. Besides, if women need money to address special needs, the community could support them; the culture of mutual aid is also common.

*“Usually*, *women have no financial income and manage their household by their husbands’ income*. *They participate in a union*, *and save money at the request of their husbands*. *Once upon time*, *I face a problem*, *my child was sick*, *and I did not have money*, *then I borrowed money from our ‘Iddir’ and saved my child’s life*. *So*, *I think ‘Iddir’ is a valuable asset for the community*.*”* (a 36-year old religious leader)

*“Social networks are important*. *We are not teaching such a wide range of health extension programs on these networks*, *but they are important*. *When there is a problem*, *we ask the chairman to provide financial support and they will do so on behalf of the association*.*”* (a 35-year old HEW)

The association in Muslim religion during *‘Mewlid’* [The Prophet’s Birthday] gathers the poor and feeds them.

*“Women and children are helped during our fasting time*. *According to our spiritual book*, *the believer contributes 25 birr out of a thousand birr income; that is saved for the poor*, *for the orphans*. *It is given to women who do not have a livelihood*. *We would distribute it to the poorest people in our ‘Mewlid’*.*”* (a 78-year old religious leader)

#### Community cohesion

*Domestic cohesion*. Some husbands supported their wives during prenatal care and childhood vaccinations in which they accompany their wives during health facility visits; organize transportation at the commencement of labor pain.

*“I have good family and relatives; they will support my wife during [her] pregnancy or childbirth*. *My brother did better things for her than I would do*. *Clothes and shoes [for the new baby] are usually provided by the families and relatives*.*”* (a 36-year old religious leader)

Another participant also described,

*“A husband loves his wife more than he loves his mother*. *No one wants his wife to be abused*.*”* (a 78-year old religious leader)

Most husbands in urban settings take care of their wives in the absence of a relative in nearby. Other family members and relatives were also responsible for the health of the mother and the baby.

*“It used to be a men’s participation program to help pregnant women*. *As they presented their experiences*, *we encountered men who cooked and baked*. *Men are more involved in hard work*, *such as washing clothes and fetching water*.*”* (a 54-year old religious leader)

On the other hand, husbands were not actively involved in community-based health services since HEWs could not find them during home visits. One HEW explained that,

*“Men’s participation is low because we do not usually find them at home*. *If we scheduled and met them during our visits*, *their attitude could have well*.*”* (a 30-year old HEW)

*Neighbourhood cohesion*. Religious leaders provided support to their neighbours regardless of their religious affiliations. They also participated in any community development activities. In ceremonies like marriage, neighbours shared responsibilities with the other.

*“As a Muslim*, *it seems wrong to build a church*. *But*, *if someone asks me to build a church*, *I contribute money for that*. *For example*, *when saint Gebriel*, *Kidane-Mihiret and Michael churches built*, *I have contributed 800 birr for each*.*”* (a 78-year old religious leader)

*“Grandmothers*, *fathers safeguard the village*. *When the young man goes to work*, *they work from home to the best of their ability*.*”* (a 38 year old religious leader)

Most children in the neighbourhood were considered as family members. This might not be possible elsewhere,

*“As it is a new neighbourhood*, *our children leave us and eat at home together; in our area*, *the child in the neighbourhood is considered to be your child*. *If you buy bread or tea*, *you will give everything equally*. *They eat their breakfast in the some house; then they eat their lunch at your house*, *and they eat dinner at the other neighbor’s house*. *This is our social life*.*”* (a 36-year old religious leader)

*“The nearest neighbour is better; if I have a relative from a distance*, *but my Christian neighbour is in trouble*, *I should give priority to my nearest neighbour*, *not my relative*.*”* (a 78-year old religious leader)

Neighbour’s relationship has both positive and negative impacts on uptake of MCH services.

*“If neighbours have satisfied with the healthcare*, *while drinking coffee*, *neighbours advise pregnant women to visit a health facility*. *In contrast*, *if someone experienced wrong things in the health care*, *he/she communicates the bad feelings to the other neighbours*.*”* (a 54-year old religious leader)

*“Traditionally*, *the community is intertwined*. *It may be on the ‘Iddir’*, *coffee ceremony or other association*. *The community is intertwined in everything*. *As a health extension worker*, *the connection between the family and the community will reduce my fatigue when I meet them together*. *To provide health education*, *and conduct drama conversations; it is good to get together to share one’s insights with one another*.*”* (a 30-year old HEW)

*Trust*. Most women in rural areas trust the community health services provided by HEWs than any other health professional. They had a positive attitude towards HEWs.

*“They [women in rural areas]) accept health information provided by us because they are familiar with us*. *If you provide medicine to them*, *people are very skeptical at this time; no one will take medicine from unknown persons*. *The community trusts me more than you*.*”* (a 30-year old HEW)

However, women in urban residences preferred hospitals since they were referred from health centers to hospitals in the absence of medical materials and health professionals.

*“Sometimes they do not trust services given in the health center because of the lack of materials and expertise*.*”* (a 36-year old religious leader)

*“Urban people underestimate health professionals working in health center*. *Although a midwife is working in both a health center and a hospital*, *they perceive that they can find a better professional at hospital*.*”* (a 35-year old HEW)

Few religious leaders thought that a social network particularly *‘Iqqub’* is not relevant and members in the networks do not trust each other.

*“I do not think ‘Iqqub’ is useful right now because the members of ‘Iqqub’ do not trust each other*. *If you see*, *court files*, *most cases are ‘Iqqub’ related*. *That is why I quit*. *It is useless right now*.*”* (a 78-year old religious leader)

Likewise, some women development groups are not functional; previously, there were training and government support to the WDA leaders. The community perceived the volunteer services provided by the WDA leaders wrongly as if they are paid workers. Therefore, it is needed to manage the wrong community’s perception towards WDA and other volunteer services.

*“Previously*, *the leaders of women development groups were given some form of refreshment training every six months*. *…now that support doesn’t continue*.*”* (a 30-year old HEW)

*“I often participate in volunteer activities and attend meetings*. *However*, *the community perceived [us] in the wrong way; they say*, *"She is working for the purpose of her own salary*.*" Just*, *we provide free service; volunteering always works with free*. *They came to my house in the morning and said*, *"Why do not you help us*? *You are receiving your salary*.*”* (FGD, a 26-year old WDA leader)

## Discussion

The purpose of this study was to explore perspectives of women, religious leaders and CHWs on the role of social capital in improving uptake of MCH services in Northwest Ethiopia. Our study identified various constructs of social capital such as membership in social networks, community mobilization, participation in social events, emotional, financial and social support, trust, and domestic and community cohesion contributing for improving the update of MCH services uptake for women and children. Women perceived that they received special care and support from close family members, relatives, neighbours, religious leaders, HEWs and other health professionals. This implies that social support, one dimension of social capital, to women was enhanced and they were motivated to visit health facilities during pregnancy and postpartum periods. Previous study conducted in Sri Lanka showed that family and neighbours provided special care and support for pregnant women [56]. In addition, the individual-level civic participation, social cohesion, or mutual exchange of social support in Japan was significantly associated with provision of immunization services [57].

In our study, most women, HEWs and religious leaders participated in social networks such as *‘Iddir’*, *‘Iqqub’* and ‘*Maheber’*. Participants attested that these networks were helpful to mobilize the community and served as a platform to encourage women or families to resume interrupted vaccinations for their children. Likewise, a previous study indicated that community-based organizations such as *‘Iddirs’* are among the most important mechanisms for the successful implementation of the multisectoral response to the problem, as they are strategically placed to facilitate community involvement and individuals tend to join *‘Iddir’* when they start families [12]. We also found that women participated in women-only networks such as women’s forums, association meetings; conferences dedicated to pregnant women were preferred networks. This could be attributed to that women-only platforms may foster discussion and share experiences with fellow women freely. In addition, the group members who accessed health information regarding MCH services could transfer messages to their neighbours. Consistent with our findings, previous studies conducted in Ghana [58], Bangladesh [9], and Kenya [59], showed that social network members were able to inform non-member women about health facility delivery.

In Ethiopia, recruited WDA leaders are expected to receive orientation on the health extension packages for 60 hours assuming that these leaders would share this health information with their network members [20]. However, in this study, we found that WDA leaders did not receive proper orientation, training and government support. As a result, WDA leaders’ knowledge of MCH became low and the community may not trust health information provided by these CHWs. Other studies have also pointed out that CHWs suffered from weak linkages with the health system, insufficient supervision, and lack of incentives, and recognition [60–64]. This might be due to lack of resilient community health system during the occurrence of conflicts in the various part of the nation. Participants in our study also mentioned that WDA leaders were heavily burdened with several activities that are not related with community health. Hence, these challenges might be a barrier for utilizing MCH services. A systematic review in other setting was also pointed out the dark side of social capital. Social capital is a double-edged phenomenon with both positive and negative effect on health [65].

Study participants mentioned sociocultural factors such as social norms, the religious belief of women and traditional practices as reasons for not seeking MCH services. For example, the restriction of out-of-home movement after childbirth hinders women and children from accessing health services. Some of the women in this study believe that they have to stay in home until priests baptize the birthing mother. In addition, the perception of the community towards adolescent pregnancy was a barrier for not commencing ANC on a timely basis. Some women also explained that childbirth in health facility is not comfortable for them in which delivery coach might expose their private bodies. Other studies also showed that privacy is greatly valued by women, yet it may be difficult to give birth in a facility due to lack of private labor wards [46, 66, 67].

Health professionals, nongovernmental organizations and religious leaders in the study area mobilize the community and create awareness about the health of women and children using various social network platforms. The relationship among members in the social networks was good that favors health education, financial support, and sharing one’s insights with one another. The community had a culture to provide emotional and social support to women although financial support is not as such common in the community. Consistent with our findings, previous studies highlighted that continuous support in childbirth may improve a number of outcomes for both mother and baby [68, 69]. Along with health care providers, religious leaders encourage women emotionally, create awareness to give birth in a health facility, clarify misperceptions of people towards vaccinations, respect pregnant women, and give due attention to children. However, other studies in Ethiopia [70, 71], Tanzania [72], and Mozambique [73], indicated that women experienced non-consented care, non-confidential care, verbal and physical abuses from health workers during childbirth. This may suggest that in addition to awareness creation in social networks, discussion forums among stakeholders including women, healthcare providers, CHWs, and religious leaders are needed to identify mechanism to solve the barriers to MCH service use.

*‘Iddir’* is a popular social network aiming to provide financial assistance for members in Ethiopia [15]. When children got sick, study participants in the study informed that they could borrow money from *‘Iddir’* to cover the medical cost of their children. In addition, the community could provide financial support to postpartum women. Consistent with our finding, a previous study conducted in Ethiopia showed that meetings of *‘Iddir’* are ideal places where women share experience; and discuss their worries. Other benefit of *‘Iddir’* include opportunities for social interaction, risk sharing and development of friendships, dispute resolution, sharing and using timely information more effectively [74]. Other studies conducted in Bangladesh [75] and Kenya [76] showed that women received significant support including pocket money to cover expenses for pregnancy care and childbirth-related services.

Study participants highlighted that a husband and other family members play a pivotal role to addresses the challenges of transportation during labor and delivery time. Qualitative evidence synthesis in LMICs also showed that the husband plays a complex role in facilitating or preventing his wife from accessing MCH services and this role varies across different contexts [45]. For example, better pregnancy outcomes have been shown when men are involved in ANC attendance, pregnancy support, and facility-based delivery [69, 77, 78]. In contrast, a husband may prohibit a facility visit due to financial or cultural constraints [79, 80].

In general, the overall attitude of women toward HEWs is positive and this cognitive social capital favors uptake of MCH services. This study also found that more people trust the community health services provided by HEWs than any other health professionals. Likewise, pregnant women in Sri Lanka have trust in the grass root level health care workers who provide informational, instrumental and emotional support during pregnancy [56]. Other studies in Ethiopia also supported our finding that women preferred to be seen by HEWs who were familiar with them rather than health workers they did not know [81, 82]. Hence, the collective cohesiveness would empower the community, and create an enabling environment for women to access health services.

### Strengths and limitations

This study has its own strengths and limitations. Using in-depth interviews and FGDs as data collection methods, and involvement of multiple study participants were opportunities to have diverse perspectives. The other strength of this study was inclusion of religious leaders who are the drivers of change at the community level due to their higher social capital. The findings would be useful in modifying cultural behaviors of social networks that negatively affect MCH services.

The study should be interpreted with its own limitations. In a patriarchal community such as Ethiopia, old men particularly often serve as opinion leaders owing from their roles as religious or community leaders. Moreover, it was not uncommon that most of the social community networks such as ‘*Iddir’* and ‘*Iqqub’* are being led by men. To reflect this, we purposely invited male participants with previous and current roles of leading these social networks to explore their perspectives on the role of social capital in improving uptake of MCH services. However, we have now noted the limitations that women participants’ views were not explored sufficiently. In addition, FGD participants who provided or received support from the social network members might not reflect their idea genuinely during discussion that leads to social desirability bias. We also relied upon the report of women, HEWs and religious leaders about husbands’ involvement and support during pregnancy, childbirth and postpartum period. In addition, the lead author (EWM) has been working in MCH research; there might be a potential bias in the research. However, the lead author and co-authors were careful not to impose their own perspectives during data collection and analysis.

## Conclusions

The social capital of women, religious leaders and CHWs plays an important role to improve the uptake of MCH services. The community has an indigenous culture of providing emotional, instrumental and social support to women through social networks. So, it would be useful to consider the social capital of family, neighborhood and community as a tool to improve utilization of MCH services. Therefore, policymakers should design innovative strategies such as people-centered health programs to engage social networks and religious leaders for improving MCH services. However, the negative experiences of the study participants including low level of knowledge and mistrust of WDA leaders, perception of the community towards adolescent pregnancy, and the social norm on restriction of women’s out-of-home movement after childbirth were barriers for uptake of MCH services. Hence, decision makers need to consider the negative effect of social capital on MCH service utilization. The regional health bureau needs to strengthen CHWs by giving optimum training for WDA, religious and community leaders to access credible health information closer to the community. Community awareness toward adolescent sexual and reproductive health services is needed. Moreover, future research should explore how continuous support by social networks to women can be best provided and challenge the social norm on restriction of women’ out-of-home movement after childbirth.

## Supporting information

S1 Appendix Term definition for local social networks in Ethiopia. (DOCX)

S2 Appendix In-depth interview guide for religious and community leaders. (DOCX)

S3 Appendix In-depth interview guide for health extension workers. (DOCX)

S4 Appendix Focus group discussion guide for women’s health development army. (DOCX)

S5 Appendix Focus group discussion guide for women. (DOCX)
