# Supplementary material for: Social capital and its role to improve maternal and child health services in Northwest Ethiopia: A qualitative study
Source: PLoS One. 2023 Apr 21;18(4):e0284592. doi: 10.1371/journal.pone.0284592 (PMC10120927; doi:10.1371/journal.pone.0284592)
Supplement: S1 Appendix — (DOCX) [file pone.0284592.s001.docx]

Appendix 1: Term definition for local social networks in Ethiopia

| **Terms** | **Description** |
| --- | --- |
| ***Iqqub*** | *‘Iqqub’* is a circular saving system in which relatives, neighbours or friends collect money to build each member’s financial capacity. In Ethiopia, ‘*Iqqub’* schemes constitute an economic institution that relies on social capital for resource mobilisation in the community. The schemes have promoted in overcoming multiple financial and non-financial constraints in their operation (Pankhurst, 2003; Aredo, 1993). |
| ***Iddir*** | *‘Iddir’* is a self-help voluntary informal association that serves as economic and social insurance at times of the death of a close family member and other crises. In the early days, an ‘*iddir’* often only had a ‘*dagna’* (judge) and perhaps a ‘*leffafi’*, a herald who blows a horn to alert members of a funeral. However, the positions of ‘*genzeb* *yazh’*, keeper of the money, and ‘*tsehafi’*, the secretary, soon became vital for administering the money and the records. Most ‘*iddirs’* have a chairman or “judge,” and nearly all have a secretary and a treasurer. More recently a range of other officers have become common, as well as subcommittees for various purposes. Some highly developed ‘*iddirs’* in urban areas have a more complex leadership structure, and a few even have their own lawyers and reinsure themselves with commercial insurance.  Significant steps in the formalization of ‘*iddirs’* include the calling of regular meetings, the keeping of minutes, and the establishment of bylaws to regulate how funds will be collected and disbursed and how fines will be assessed.Individuals tend to join *‘iddirs’* when they start families; however, younger people who are not married are also active members of many ‘*iddirs’*, and youth ‘*iddirs’* are also becoming popular. The main reasons for people not being members of ‘*iddirs’* are that they are new settlers (not well established) in a particular area or are not able to pay contributions. Many ‘*iddirs’* have provisions for members who face economic problems and are unable to pay contributions. These members will be considered “pensioned” by the ‘*iddir’* and still receive all the benefits due to other members without paying contributions. Their influence is so strong that a person who does not belong to an *iddir* is usually considered an outcast and a disgrace to his or her family. |
| ***Senbetie*** | *‘Maheber’* and *‘Senbetie’* are socio-religious associations that hold gatherings, with spiritual and social functions named after saints. In *‘Senbetie’*, members rotate in bringing food and drinks to be consumed by the priests after Holy mass each week around the church. Both husbands and wives are responsible and participate equally. |
| ***Maheber*** | *‘Meheber’* refers members seek to honor the saints by gathering at a member’s house on a saint’s day every month, with the (rotating) host providing food for the guests. In case of *‘Maheber’*, men and women may have different memberships in the name of different saints. Usually, men belong to St. Michel (on 12th), St. Gabriel (on 19th) and St. George (on 23rd) *‘Maheber’*. Likewise, women belong to St. Mary (on 21st) and Kidane Mihiret (on 16th) *‘Maheber’* (the dates are in Ethiopian calendar). |
