# Supplementary material for: Social capital and its role to improve maternal and child health services in Northwest Ethiopia: A qualitative study
Source: PLoS One. 2023 Apr 21;18(4):e0284592. doi: 10.1371/journal.pone.0284592 (PMC10120927; doi:10.1371/journal.pone.0284592)
Supplement: S2 Appendix — (DOCX) [file pone.0284592.s002.docx]

# Appendix 2: In-depth interview guide for religious and community leaders

**Code** ________________

**Time started**_____________

**Background information of interviewees**

- Name of district_____________________
- Residence □ Rural □ Urban
- Age in years _______________
- Religion___________________
- Marital status_______________
- Educational status ___________________
- Role in the community/religious institution _________________

1. What health services do you think mothers and children in your community seek?

*[Probe: What type of health facilities (hospital/ health center/health post) is available in your locality? Could women access antenatal, delivery, and postnatal care services in that facility? Could children access immunization services? What is your view on the importance of these maternal and child services to women and children?]*

1. Do you have a network that you meet with other people in your community? If so, do you discuss health issues with them?

*[Probe: Which formal social networks (Eddir, Eqquib, Mahiber, Senbetie, Wonfel, or Debo) are you commonly attending in your community? In which type of social networks do you actively engage? Can you describe your roles in that social network [member or chairperson, etc.]? Do these networks talk/discuss what services are available for mothers and children in your community (e.g. antenatal care services, child immunisation, and birth at health facility)?*

1. As a community/religious leader, do you believe that any of these social networks/associations are beneficial for health?

*[Probe: What is your experience in relation to how social associations help to disseminate/communicate the benefit of accessing health services (antenatal care, delivery, postnatal care, and child immunization services)? Have you involved in any mobilization for women and children health campaigns so far? If so, give example. Do you think that community/religious leaders can help women to give birth at the health facility rather than at home?]*

1. Do you perceive that you have good relationships with community volunteers/traditional birth attendants, health extension workers, and health development army to promote the health of mothers and children?

*[Probe: How do you express the communities trust to services/information provided by health extension workers and health care providers in health centers? Do you think that mothers trust the information they receive from their social network? Does community health program result in any behavioural change (e.g. Can you mention any change that has been malpracticed in the community)?]*

1. How do you work together with other community members? Have you attempted to address a problem or common issue of the village?

*[Probe: Whom do you have discussed about the development of your village? Have you discussed with Leaders of the Village, Religious leaders, Political Leaders, Government officials, or Personnel from non-governmental organization? Do you think that involving in social groups could influence uptake of maternal and child health services? How?]*

1. Do you believe that the informal social network such as neighbors and families (e.g sisters, mothers, grandmothers) help women to receive health services? Is so, in what way?

*[Probe: Do families or neighbors look after children left at home or cattle for women to visit health services? Would there be any financial or moral support to be obtained?]*

1. Do you also perceive that social networks/associations have negative role for women and children not to receive health services?

*[Probe: Are there circumstances where women in your community told not to visit health services? Can you give me examples? Do you think existing social network (e.g. Eddir) propagate negative socio-cultural practices that discourage women and children to access healthcare?]*

1. Do you think traditional beliefs, religious practices, and cultural norms affect mothers to use care during pregnancy, delivery, and the postpartum period in your community?

*[Probe: Explain how and why? Can you tell us about the traditional practices and beliefs during pregnancy, delivery, and the postnatal period in your community? Can you explain the beliefs of the community towards immunization? Could individuals, including other pregnant women, older people, partners, and relatives (parents) influence maternal vaccination during pregnancy? Do they influence childhood vaccination? Do they trust vaccines? Are there any rumors about vaccination? If so, what are these? Do women use local remedies instead of modern medicines (vaccines)?]*

1. In your community, how do women prepare for birth? What birth preparedness-related services are found in your community?

*[Probe: Where do women prefer to give birth and to be assisted? Why all women do not seek delivery care at health facilities? What is the value of traditional birth attendants in the community? Do you encourage women to deliver in the facility? Are you familiar with the maternal waiting area? What are the factors that discourage pregnant mothers not to stay in maternal waiting area before immediately close to their delivery? What are the solutions you suggest?]*

1. What preparations are found in your community for emergencies related to maternal and child health?

*[Probe: Do people make preparations? What barriers hinder such readiness? What possible solutions do you suggest to curb such problems? How do women get support for their social networks, including ‘Eqquib’ and ‘Mahiber’?]*

1. In your community, who will make the final decision on place of birth and birth assistance? Why?

*[Probe: What is the extent of husband`s, mother-in-law’s, and grandmother’s involvement in decisions on the use of MCH services? What happens if relatives/husbands disapprove of seeking the care? Is there any social taboo regarding male involvement in delivery service?]*

1. What are problems in your community that hinder women from getting health services during pregnancy, childbirth, and postpartum period?

*[Probe: Is there any fear and misperceptions about medical interventions? Do you think that birth practices and care at health facilities are culturally acceptable?]*

1. Is there anything you would like to add

[Probe: What should be improved regarding the continuum of maternal and child services?]

Time ended____________________

Thank you for your time and great participation
