# Supplementary material for: Social capital and its role to improve maternal and child health services in Northwest Ethiopia: A qualitative study
Source: PLoS One. 2023 Apr 21;18(4):e0284592. doi: 10.1371/journal.pone.0284592 (PMC10120927; doi:10.1371/journal.pone.0284592)
Supplement: S3 Appendix — (DOCX) [file pone.0284592.s003.docx]

# Appendix 3: In-depth interview guide for Health Extension Workers

**Code** ________________

**Time started**_____________

**Background information of interviewee**

- Name of district_____________________
- Residence □ Rural □ Urban
- Name of health center and health post______________
- Age of interviewee__________________
- Marital status_______________
- Responsibility of the HEW__________________
- Total number of years/months working_________________

**Interview Guide**

1. How do you schedule your time for maternal and child health services and other health extension programs?

*[Probe: How many days of the week are allocated for you to provide the maternal and child health services? What is your role as health extension worker to promote the uptake of maternal and child health services? Could you please give a detailed list of maternal and child health services you provide for the community?]*

1. In the community you are working with, how do you describe the social networking between community members, families, or parents?
2. In your opinion, do you think existing social networks are helpful for promoting maternal and child health services?

*[Probe: Have you attended any of social gatherings (e.g. Eqqub, Idder, Senbete) to disseminate health information, create awareness about available health services for mothers and children in your facility? To what extent do you leverage these networks for you to disseminate health information?*

1. What do you think are the common problems in your community that hinder pregnant women from getting health services during pregnancy, childbirth/labor, and postpartum period?

*[Probe: What are the common reasons that make women not prefer to follow antenatal care services? What are the reasons for high dropout rate? What possible solution do you suggest to curb such problem?]*

1. How do you work together with leaders of the village, religious leaders, political leaders, government officials, personnel from nongovernmental organizations, health development army, opinion leaders, model households, and other community members to promote the health of mothers and children?

*[Probe: Have you spoken with anyone about the development of your village? Do you have a routine meeting with health development armies? In what ways do residents, community, and religious leaders participate in maternal and child health activities? How do you involved men during a household visit and health education concerning maternal and child health?]*

1. How often do higher officials (health center officers) visit your community related to maternal and child health services?

*[Probe: What do they do? What does the guideline say about the visit? How do you get feedback from health center officers or district health offices regarding maternal and child health services? Does the feedback have values?]*

1. Do you think the members of the community in your workplace trust the information provided by health extension workers?

*[Probe: Do you think your neighbors in your village trust health information provided by you? Do you think people residing in your village trust health information related to antenatal care, delivery, postnatal care and vaccination?]*

1. How do you think social networking needs to be used as an opportunity to create awareness about mothers and children health services and to increase mothers and children access to health services?

*[Probe: In your opinion, what should be improved regarding to antenatal care, delivery, postnatal care and vaccination]*

1. Is there anything you would like to add

Time ended __________________

Thank you for your time and great participation
