# Supplementary material for: Social capital and its role to improve maternal and child health services in Northwest Ethiopia: A qualitative study
Source: PLoS One. 2023 Apr 21;18(4):e0284592. doi: 10.1371/journal.pone.0284592 (PMC10120927; doi:10.1371/journal.pone.0284592)
Supplement: S4 Appendix — (DOCX) [file pone.0284592.s004.docx]

# Appendix 4: Focus group discussion guide for Women’s Health Development Army

| **Identification** | | |
| --- | --- | --- |
| 1 | ID | **____________________________** |
| 2 | Area Identification | **____________________________** |
| 3 | Name of Woreda | **____________________________** |
| 4 | Name of Kebele | **____________________________________** |
| 5 | Name of moderator | **_______________________________** |
| 6 | Name of note taker | **_________________________________** |
| 7 | Date of discussion | **_______________________________** |
| 8 | Start time: | **______:________** |
| 9 | End time: | **____:______** |

**Interview Guide**

1. How do you schedule your time for maternal and child health services and other health extension programs?

*[Probe: How many days of the week are allocated for you to provide the maternal and child health services? What is your role as health development army to promote the uptake of maternal and child health services? Could you please give a detailed list of maternal and child health services you provide for the community?]*

1. In the community you are working with, how do you describe the social networking between community members, families, or parents?
2. In your opinion, do you think existing social networks are helpful for promoting maternal and child health services?

*[Probe: Have you attended in any of social gatherings (e.g. Eqqub, Idder, Senbete) to disseminate health information, create awareness about available health services for mothers and children in your facility? To what extent have you leveraged these networks to disseminate health information?*

1. What do you think are the common problems in your community that hinder pregnant women from getting health services during pregnancy, childbirth/labor, and postpartum period?

*[Probe: What are the common reasons that make women not prefer to follow antenatal care services? What are the reasons for high dropout rate? What possible solutions do you suggest to curb such problems?]*

1. Do you think the members of the community in your workplace trust the information provided by women developmental army?

*[Probe: Do you think your neighbors in your village trust health information provided by you? Do you think people residing in your village trust health information related to antenatal care, delivery, postnatal care and vaccination?]*

1. How do you think social networking needs to be used as an opportunity to create awareness about mothers and children health services and to increase mothers and children access to health services?

*[Probe: In your opinion, what should be improved regarding to antenatal care, delivery, postnatal care and vaccination?]*

1. Who supervise your efforts and actions to safe the mothers and newborn babies in your community?

*[Probe: Did health extension workers visit your home last year? How frequently they visit your home? Do you think that you have adequate knowledge to provide promotive, and preventive maternal and child health services? Are you satisfied by your actions so far? Why or why not?]*

1. Do you feel that you have a sense of belonging to this village?

*[Probe: Do you think that the majority of people in your village would try to take advantage of you if they got the chance? Do you think the majority of people in this village generally have good relationships with each other?]*

1. Overall, do you think the leaders of your village or members/leaders of the social network can be trusted?

*[Probe: Do you think your neighbors in your village can be trusted? Do you think people whom you are not familiar with and residing in your village can be trusted? What about your trust in health care providers?* *Could you tell me how the community has trusted the women developmental leaders?* *How do you see the relationship and communication among women developmental army members, network leaders and 1 to 30 leaders?]*

1. Is there anything you would like to add?

*[Probe: In your opinion, what should be improved regarding antenatal, delivery, postnatal, and immunization services?]*

Thank you for your time and great participation
