# Supplementary material for: Social capital and its role to improve maternal and child health services in Northwest Ethiopia: A qualitative study
Source: PLoS One. 2023 Apr 21;18(4):e0284592. doi: 10.1371/journal.pone.0284592 (PMC10120927; doi:10.1371/journal.pone.0284592)
Supplement: S5 Appendix — (DOCX) [file pone.0284592.s005.docx]

# Appendix 5: Focus group discussion guide for women

| **Identification** | | |
| --- | --- | --- |
| 1 | ID | **____________________________** |
| 2 | Area Identification | **____________________________** |
| 3 | Name of Woreda | **____________________________** |
| 4 | Name of Kebele | **____________________________________** |
| 5 | Name of moderator | **_______________________________** |
| 6 | Name of note taker | **_________________________________** |
| 7 | Date of discussion | **_______________________________** |
| 8 | Start time: | **______:________** |
| 9 | End time: | **____:______** |

| **Participant Demographic Intake Sheet** | | | | | | |
| --- | --- | --- | --- | --- | --- | --- |
| 1 | Participant code |  |  |  |  |  |
| 2 | Age |  |  |  |  |  |
| 3 | Religion |  |  |  |  |  |
| 4 | Marital status |  |  |  |  |  |
| 5 | Are you employed? (Yes/No) |  |  |  |  |  |
| 6 | Educational level |  |  |  |  |  |
| 7 | Gravidity |  |  |  |  |  |
| 8 | Parity |  |  |  |  |  |
| 9 | Place of delivery in last pregnancy (facility/home) |  |  |  |  |  |

**Interview guide**

1. Would you please describe about the health services of mothers and children?

*[Probe: Do you think health care for mothers and children is important? Who is providing health services for mothers and children in your area? How do family members or neighbors support you in caregiving for children?]*

1. How pregnant mothers are going for antenatal care in your village or kebele?

[Probe: Do you tell me about antenatal care and its importance? Where do they prefer to go for antenatal care? Why?]

1. Would you describe problems you have experienced related to the utilization of antenatal, delivery, and postnatal care?

*[Probe: What are the obstacles in accessing the care? What are the reasons for not getting it? What are the reasons for your interruption/discontinuation of getting the care? What do you say about your confidence on skilled birth attendants, and about their respect and compassion r the traditional beliefs of the women]*

1. Now let’s discuss new-born’s care; how the new-borns are receiving care?

*[Probe: What is going to be done for the newborn? When? Where? By whom? Why? What do you feel or suggest important care to the newborn and how? What about immunization, when and which vaccination?]*

1. In your community, would you describe your participation in any social groups?

*[Probe: Have you been a member of any social groups including microcredit group/ ‘Eqquib’, self-help group, religious group/’Mahiber’/’Senbetie’, village council, and any other women group? Do you think that participating in social groups could help to get information for the health of mothers and children?*

1. Would you perceive that being a member of a certain social network would have health benefits in addition to why the network is established? Can you explain?

*[Probe: Have you received any support (emotional or financial) from any of the networks/groups during pregnancy or ill health? Could you describe the support you have received from your social network? E.g. Pooling finance for supporting transportation and payment for health services]*

1. How do you describe the support you obtain from family member, relatives, neighbors, village leaders, religious leaders, and personnel from the non-governmental organizations?
2. Do you feel that you could have a good feeling of belonging to this social network?

*[Probe: Do you think that the majority of people in your village would try to take advantage of you if they got the chance? Do you think the majority of people in this village generally have good relationships with each other?]*

1. Overall, do you think the leaders of your village or members/leaders of the social network can be trusted?

*[Probe: Do you think your neighbors in your village can be trusted? Do you think people whom you are not familiar with and residing in your village can be trusted? What about your trust in health care providers?]*

1. Now we are on the way to windup, but before we do so, anything to be added or you anything/message you need to pass to anybody?

*[Probe: what should be improved regarding maternal and child health services?]*

*Thank you for your time and great participation*
